# Synergistic Effect of Metformin and Lansoprazole Against Gastric Cancer through Growth Inhibition

**DOI:** 10.7150/ijms.82407

**Published:** 2023-04-17

**Authors:** Hsiao-Wei Kao, Kuo-Wang Tsai, Wen-chang Lin

**Affiliations:** 1 Institute of Biomedical Sciences, Academia Sinica, Taipei, Taiwan, R.O.C.; 2 Institute of Biomedical Informatics, National Yang Ming Chiao Tung University, Taipei, Taiwan, R.O.C.; 3 Department of Research, Taipei Tzu Chi Hospital, Buddhist Tzu Chi Medical Foundation, New Taipei City, Taiwan, R.O.C.

**Keywords:** Metformin and Lansoprazole, Synergistic Effect, Gastric Cancer

## Abstract

Cancer has been linked to metabolic disorders and diverse gene mutations. Metformin, which is widely used to treat type 2 diabetes, inhibits the growth of cancer cells in animal models. Here we investigated the effects of metformin on human gastric cancer cell lines. We also investigated the synergistic anticancer effect of metformin and proton pump inhibitors. Lansoprazole, a proton pump inhibitor, is effective for treating gastroesophageal reflux disease. Our results revealed that metformin and lansoprazole can significantly inhibit cancer cell growth in a dose-dependent manner by suppressing cell cycle progression and inducing apoptosis. Low concentrations of metformin and lansoprazole have a synergistic effect on AGS cell growth inhibition. In summary, our findings suggest a new and safe treatment protocol for treating stomach cancers.

## Introduction

Cancer is now considered to be caused by various oncogenic gene mutations or defects in metabolic pathways 1-3. In the 1920s, Dr. Otto Warburg discovered tumor-related metabolism alterations. His Warburg effect hypothesis on malignant tumor cells suggests that rapid glucose uptake and lactate secretion contribute to the rapid cell growth and macromolecular synthesis requirements of cancer cells 4, 5. Furthermore, heterogeneity is prevalent within cancer cells, and distinct genetic alterations can be observed in diverse populations of cancer cells 1, 6. This complicates the identification of cancer-targeting agents for specific mutations in distinct cancers. Multiple oncogenic pathways may converge on essential metabolic pathways to support cancer cell survival and proliferation 7. Different subpopulations of cancer cells demonstrate common metabolic pathways, therefore, it is proposed that treatments targeting cancer cell metabolism may be a novel approach for treating cancer. Furthermore, the chemotherapy resistance of recurrent tumor cells is often the leading cause of cancer treatment failure. Tumor resistance often resulted from additional genetic mutations altered from the original mutated sites 8. Thus, targeting common metabolic pathways may be an alternative treatment approach.

Metformin is a drug of the biguanide class, which is commonly prescribed for type 2 diabetes. Recent reports have indicated that metformin may have anticancer effects against breast, pancreas, colon, and prostate cancers 9, 10. Numerous meta-analyses have demonstrated that in addition to its direct anticancer effects, metformin reduces cancer incidence by 30%-50% 11. Many in vitro studies have demonstrated that metformin was able to reduce xenograft tumor burden without the severe side effects typically caused by cytotoxic chemotherapeutic agents 12, 13. Compared with other antidiabetic biguanides, metformin is inexpensive, harmless, and has minimal side effects. Many studies have demonstrated that combining metformin with other anticancer drugs can further improve its anticancer efficiency. The combination of metformin with small molecular inhibitors (gefitinib, sorafenib, or everolimus) and antibodies induces tumor cell death by blocking signal transduction 14, 15. Metformin exhibits significant synergism with these drugs in the treatment of lung cancer and bladder cancer 14.

Proton pump inhibitors (PPIs) target the H+/K+-ATPase protein in gastric parietal cells. They are widely prescribed for acid-related diseases, such as gastroesophageal reflux disease (GERD), gastric ulcer, and duodenal ulcer 16. According to previous research, PPIs induce apoptosis in gastric cancer cells and B-cell tumors 17. Several reports have demonstrated that PPIs inhibit tumor cell growth and development 18, 19. Therefore, in this study, we investigated the combination of metformin and lansoprazole for the treatment of gastric cancer cells.

## Material and methods

### Cell lines and cell culture conditions

Human gastric cancer cell (AGS) was purchased from the Food Industry Research and Development Institute, Hsinchu, ROC. The cells were maintained in Dulbecco's Modified-Minimal Essential Medium supplemented with 1% penicillin/streptomycin solution (Thermo Fisher Scientific, Inc., Wilmington, DE, USA) and 10% fetal bovine serum (Biological Industries, Inc., Israel) in a 37 °C incubator with 5% CO_2_. For drug treatment, the cells were cultured in Dulbecco's Modified-Minimal Essential Medium without glucose (Thermo Fisher Scientific, Inc., Wilmington, DE, USA) supplied with 5 mM glucose (Sigma-Aldrich, St. Louis, Missouri, USA). After overnight incubation, the cells were treated for additional 3 days with either 1 mM metformin (dissolved in PBS [MCE, NJ, USA]), 10 μM lansoprazole (dissolved in DMSO [Santa Cruz Biotechnology, Inc., Dallas, USA]), or a combination of both drugs.

### Drug treatment and synergism calculation

The AGS cells were seeded at a density of 5000 cells/well in a 96-well plate and maintained overnight in low-glucose medium; subsequently, they were treated for 3 days with varying concentrations of lansoprazole (0, 3, 10, 30, and 90 μM) and metformin (0, 0.1, 0.3, 1, 3, and 9 mM). Finally, the WST-1 assay was performed according to the manufacturer's protocol (Roche, Inc., Basel, Switzerland). We used the SynergyFinder package 20 to calculate the combination index (CI), which reflects the type of drug interaction.

### Proton pump inhibitor treatment

The cells were seeded at 10,000 cells/well in a 12-well plate in low-glucose medium. After overnight incubation, 10 μM PPIs were added with or without 1 mM metformin for 3 days.

### Crystal violet assay

After drug treatment, culture medium was removed from the 12-well plate and were washed once with warmed PBS at room temperature. We then added 500 μL/well of crystal violet solution (2% EtOH + 0.1 g crystal violet [Sigma-Aldrich, St. Louis, Missouri, USA] in 50 mL H_2_O), and the plates were incubated for 10 min at room temperature. The plates were washed twice with tap water and dried overnight. We then added 1 mL of 1% SDS solution to solubilize the stain by agitating the plates on an orbital shaker to completely dissolve the stain. Finally, we transferred 100 μL/well of dissolving solution into the 96-well plate, and absorbance was measured at 570 nm.

### Colony formation assay

The cells were seeded in a 6-well plate at a density of 10000 cells/well. After overnight incubation, 1 mM metformin, 10 μM lansoprazole, or the combination of the drugs was added. After 4 days, fresh medium and drug were replaced. After 8 days, colony formation was measured through the crystal violet assay.

### Cell cycle experiments

The cells were seeded in a 10-cm culture dish. After 3 days of drug treatment, the cells were collected and resuspended in 0.5 mL PBS, and 4.5 mL of pre-cold 70% ethanol was then added. The cells were fixed at -20 ℃ for at least 2 h before being centrifuged at 300 × g for 5 min. The cell pellet was suspended in 1 mL PBS, and 50 μL of the cell solution was then diluted into 450 μL of PBS in a 1.5-mL Eppendorf tube. The Attune NxT Flow Cytometer was used for determining cell numbers. We suspended 2.5 × 10^5^ cells in 1 mL of PBS, and the cell pellet was suspended in 1 mL of DAPI/Triton X-100 staining solution as per the labeling protocol 21. The suspended cell pellet was maintained in the dark for 30 min at 37 ℃. The results of cell cycle were analyzed using the flow cytometer and FlowJo (v7).

### Apoptosis assay

The cells were seeded in a 10-cm culture dish. After 3 days of drug treatment, the cells were resuspended in 200 μL of binding buffer, and the apoptosis assay was then performed with the Apoptosis Detection Kit (Thermo Fisher Scientific, Inc., Wilmington, DE, USA). The flow cytometer was used to analyze the apoptosis results.

### Cell morphology

To observe the real-time images of the cells after drug treatment, we captured time-lapse images using HoloMonitor M4 (Phase Holographic Imaging AB, Lund, Sweden). The cells were seeded in a 6-well plate at 5000 cells/well and incubated overnight. On the next day, the plate with hololid were placed on the HoloMonitor M4 after the drugs were added. The cell images were recorded every 20 min for 72 h. Finally, the holographic images were analyzed using the HoloMonitor M4 application program.

### Western blot analysis

The cells were seeded in a 10-cm culture dish. After 3 days of drug treatment, the cell pellet was collected. Total cell lysates were extracted with RIPA buffer (50 mM Tris-HCl at pH 8.0, 150 mM NaCl, 1% NP-40, 0.5% deoxycholic acid, and 0.1% sodium dodecyl sulfate). Total proteins were separated using 6%-10% gradient sodium dodecyl sulfate-polyacrylamide gel electrophoresis and then transferred to nitrocellulose filter membranes (Millipore, Billerica, USA). After transfer, the membranes were blocked with blocking buffer (PBS-Tween containing 5% skim milk) at room for 1 h temperature and incubated with primary antibodies overnight at 4 °C. Supplementary Table 1 lists the antibodies used in this study. Subsequently, the membranes were incubated with horseradish peroxidase-conjugated secondary antibody. Finally, the protein blots were visualized with WesternBrightTM ECL (Advansta Inc., USA) and detected with the BioSpectrum 500 Imaging System (Thermo Fisher Scientific Inc., Waltham, MA, USA).

## Results

### Effect of glucose concentration on drug sensitivity

**Figure 1:**
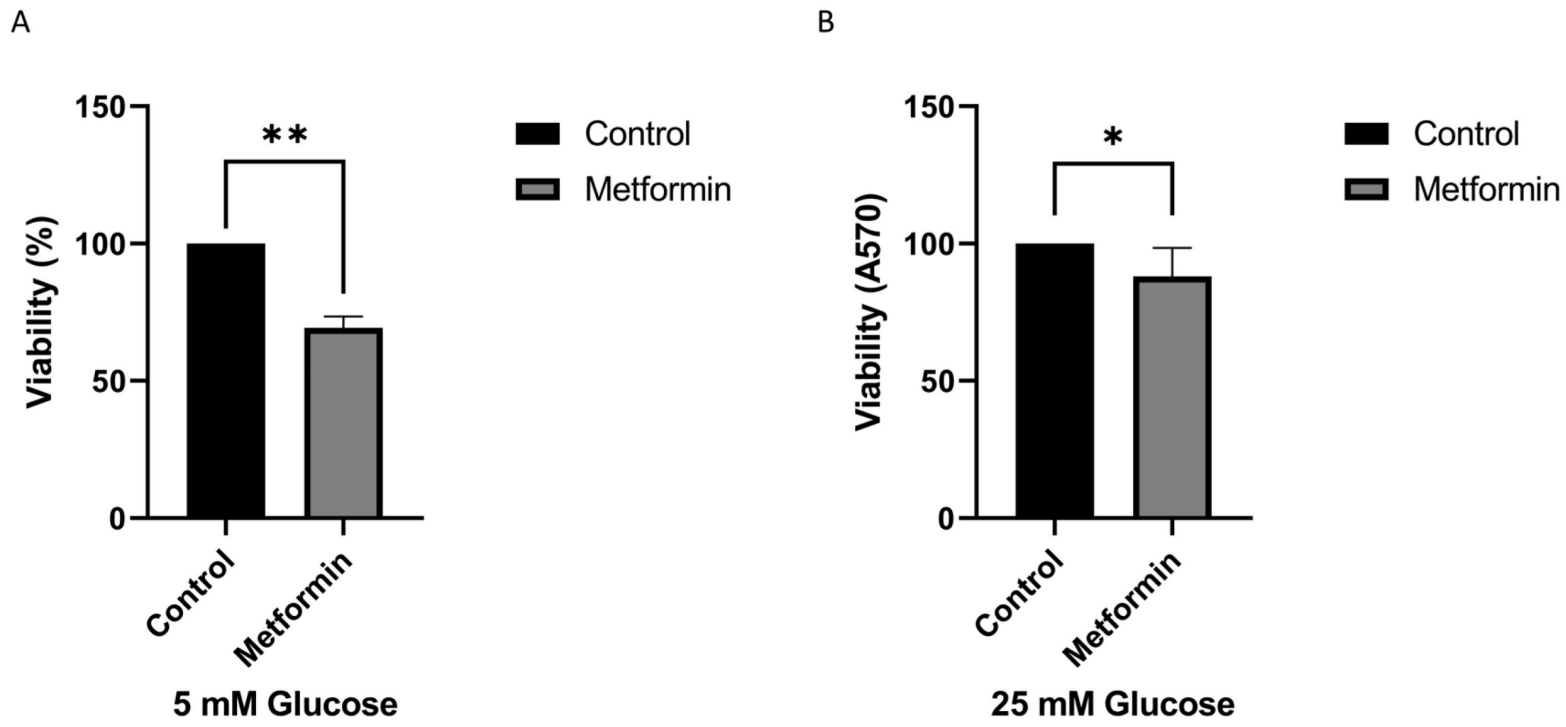
High concentrations of glucose increase drug resistance of gastric cancer cells. The AGS cell line was treated with metformin (Metformin: 1 mM) in a different concentration of glucose for 3 days. A. 5 mM, B. 25 mM of glucose. The crystal violet assay was used to calculate the viability. The data was presented as the mean with SD (**P<0.01; *P<0.1).

In this study, we examined the anticancer effects of metformin and PPI. The normal range of the blood glucose concentration is 3.9-7.1 mM. The average fasting plasma blood glucose level in humans is approximately 5.5 mM 22. Commercial medium formulations usually contain a higher concentration of glucose (25 mM) than that in blood. Because metformin is involved in regulating the physiological levels of glucose, we first assessed the effect of the cell viability under 5 and 25 mM glucose concentrations. As shown in Figure 1, we found that treatment with metformin significantly inhibited the growth of gastric cancer cells. Interestingly, metformin was more effective in inhibiting gastric cancer cell growth in low glucose concentration medium than in high glucose concentration medium (Figure 1A and 1B). In summary, our findings suggest that higher glucose concentrations may mitigate the inhibitory effect of metformin on cancer cell growth. Based on the data, follow-up studies were conducted using low-glucose medium (5 mM) with a glucose concentration closer to physiological *in vivo* glucose concentrations.

### Combined treatment of PPI and metformin

**Figure 2:**
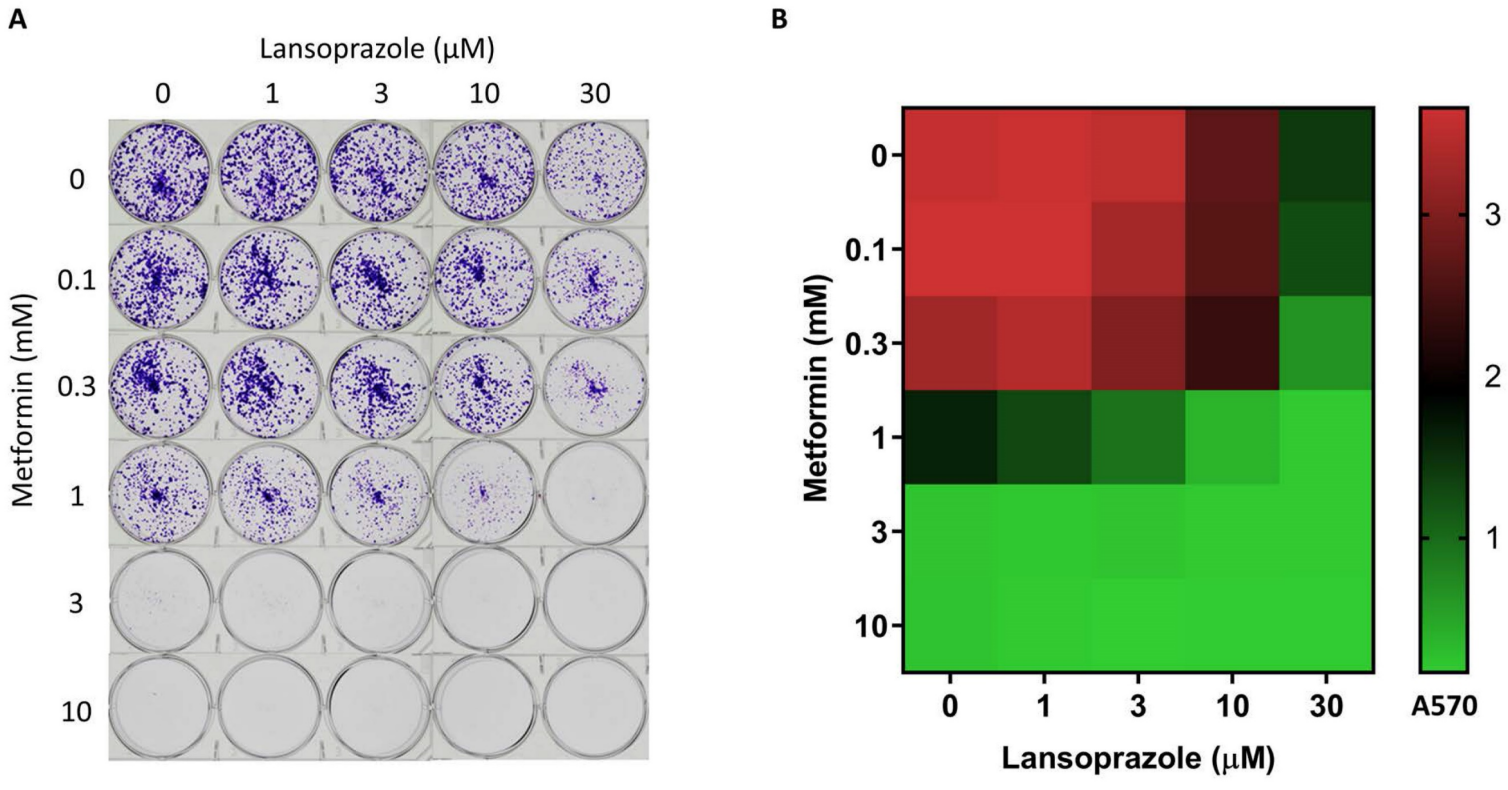
Colony formation assay. A. The AGS cell line was treated with various concentrations of metformin and lansoprazole for 8 days. The cells were cultured in 6-well plates and stained with crystal violet. B. Heat map of the AGS colony formation assay.

To examine the anticancer effects of metformin and PPI, we treated the AGS cells with varying concentrations of metformin, lansoprazole for 3 days. Metformin and lansoprazole inhibited AGS cell growth in a dose-dependent manner (Figure S1. A and B). PPIs and calcium channel inhibitors have therapeutic potential for various human cancers 23-25. We examined the inhibition effect of PPIs and calcium channel inhibitors on the AGS cells and discovered that PPIs alone had a minor effect on the AGS cells, whereas calcium channel inhibitors did not affect the AGS cells. When combined with metformin, the inhibition effect of PPIs was more significant than that of calcium channel inhibitors when combined with metformin. These findings revealed that the combination of metformin and PPIs had synergistic effects on inhibiting AGS cell growth. Among PPIs, lansoprazole has the strongest synergistic effect with metformin in our result (data not shown); thus, we chose it for further research. Further examination of the effect of lansoprazole on inhibiting AGS cell growth indicated that the viability of the AGS cells was significantly suppressed by high concentrations of lansoprazole (>30 µM), whereas the viability of the AGS cells was slightly suppressed by low concentrations of lansoprazole (<10 µM) (Figure S1B). Combined with metformin, low concentrations of lansoprazole exhibited a synergistic effect on inhibiting AGS cell growth (Figure S1C). The long-term treatment effects on the AGS cells were evaluated through the colony formation assay (8 days). We also examined varying combinations of metformin and lansoprazole in the long-term treatment groups. To inhibit the formation of AGS colonies, 30 µM lansoprazole with 1 mM metformin was found to be the optimal combination (Figure 2A and B). The HoloMonitor M4 captured a time-lapse video that demonstrated the slower growth rate of combined drug treatment (Video S2). These findings indicate that the combination of metformin and lansoprazole inhibits the growth of the AGS cells.

### Synergistic effects of lansoprazole and metformin

SynergyFinder is a web application for analyzing dose-response matrix data for drug combinations. Using the SynergyFinder package, we calculated the combination index (CI), which was used to determine the type of interaction between the combined drugs. A synergistic effect was found for the AGS cells treated with 10 µM lansoprazole and 1 µM metformin (CI = 0.57) (Figure S3). However, neither short-term (3 days) nor long-term (8 days) drug treatment resulted in significant alterations in cellular morphology (Figure S4).

### Cell cycle modulation after lansoprazole and metformin treatment

**Figure 3:**
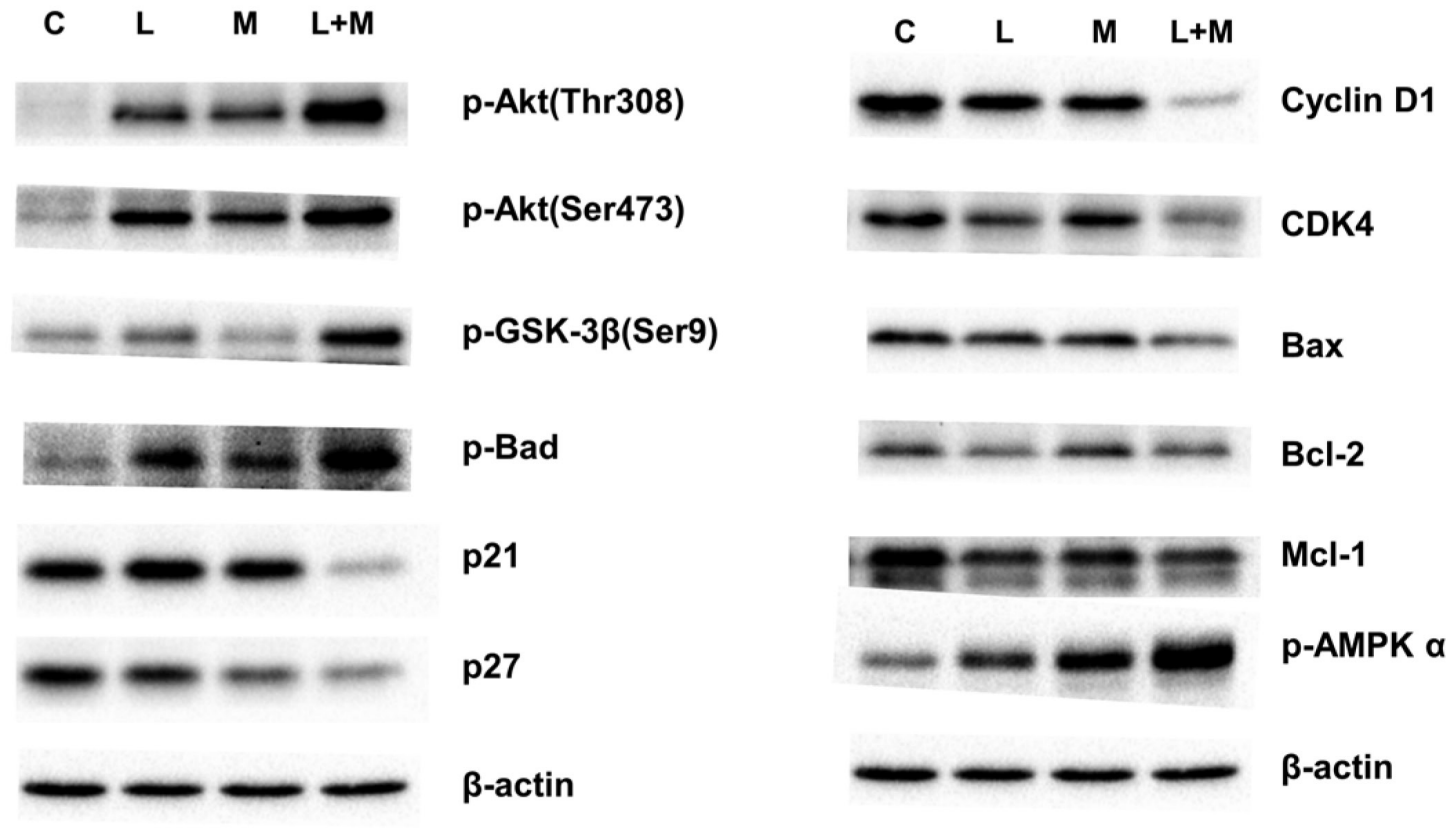
Western blot analysis. The AGS cell line was treated with metformin (M: 1 mM), lansoprazole (L: 10 μM), and combined drugs L+M (L: lansoprazole 10 μM; M: metformin 1 mM) for 3 days. Western blot was used to display the protein levels of various drug treatments.

To elucidate the effects of drug treatment on cell cycle progression, the AGS cells were treated with the drugs for 3 days; the cells were then stained with DAPI, and their cell cycles were analyzed using the flow cytometer. Combined lansoprazole and metformin treatment decreased the proportion of cells in the G1 and G2 phases and increased the proportion of cells in the S phase (Figure S5). We further examined the genes involved in cell cycle regulation. Western blot analysis revealed that Akt protein was highly activated, phosphorylating GSK-3β (Ser9) and thereby promoting cell survival. Furthermore, the low protein expression of p21 and p27 promoted cell cycle progression to the S phase. The phosphorylation of GSK-3β (Ser9) induced the phosphorylation of BAD on the Ser136 site, thereby protecting cells from apoptosis (data not shown). The decreased levels of cyclin D1 maintained the cells in the S phase in preparation for DNA synthesis (Figure 3, Figure S6 and 7). Therefore, combined drug treatment induces the cell survival pathway involving Akt/GSK-3β/BAD proteins and increases the proportion of cells in the S phase.

### Apoptosis induced by drug treatment

**Figure 4:**
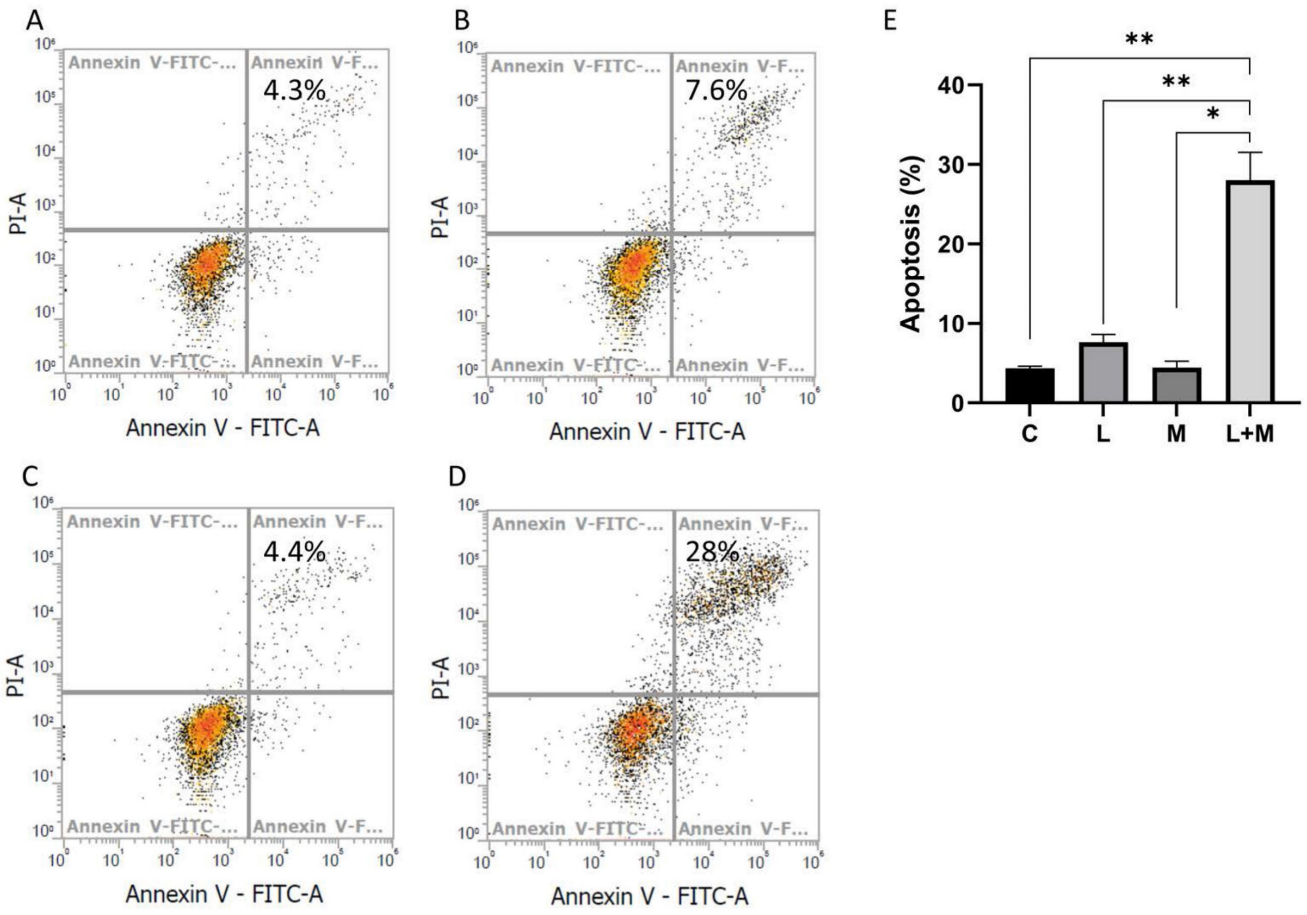
Apoptosis assay. The AGS cell line was treated with drugs for 3 days, stained with annexin V and propidium iodide, and then analyzed using the flow cytometer. A. medium only, B. lansoprazole (L: 10 μM), C. metformin (M: 1 mM), D. L+M (L: lansoprazole 10 μM; M: metformin 1 mM), and E. The data was presented as the mean with SD (**P<0.01; *P<0.05).

In addition to growth inhibition, the possibility of induction of cell death by combined drug treatment was examined. We evaluated apoptosis in AGS cells treated with metformin and lansoprazole. After drug treatment, the cells were stained with Annexin V and propidium iodide and were analyzed using the flow cytometer. The percentage of apoptosis-positive cells in the control, metformin, lansoprazole, and combined drug groups was 4.3%, 7.6%, 4.4%, and 28%, respectively (Figure 4A, B, C, D). The subsequent Western blot revealed low protein levels of cyclin D1 and CDK4, indicating a decrease in cell cycle progression. Combined drug treatment induced BAD activation, and the low expression of Bcl-2, BAK, and MCL-1 supports the notion that drug treatment also induces intrinsic apoptosis (Figure 4A, B, C, D and Figure S6 and 7). Thus, drug treatment induced intrinsic apoptosis and decreased cell cycle progression in the cells.

## Discussion

PPIs can inhibit V-ATPase, the enzyme that regulates the pH gradients in cancer cells. High V-ATPase levels are associated with metastasis and multiple drug resistance 26, 27. Some studies have proved that the induction of autophagy can promote resistance to cancer therapy, which can be inhibited by PPIs 28, 29. Man *et al*. proved that lansoprazole improves the distribution and activity of doxorubicin in solid tumors, thereby enhancing its therapeutic effects 15.

Metformin is widely used in the treatment of type 2 diabetes. Many studies have demonstrated that metformin has anticancer effects in patients with ovarian, breast, prostate, and colorectal cancers 30, 31. Its anticancer effects involve AMPK-dependent and AMPK-independent pathways 32. For the AMPK-dependent pathway, metformin inhibits cell growth through G0/G1 cell cycle arrest and the induction of autophagy in lymphoma cells. It also inhibits cell mitosis and proliferation in carcinoma tissues and cancer cell lines 27. For the AMPK-independent pathway, metformin inhibits the cell cycle in the G0/G1 phase without inducing apoptosis or decreasing the cyclin D1 protein level in prostate cancer cells 33. When combined with other drugs, metformin exhibits a synergistic effect to treat cancers 34. Morgillo *et al*. discovered that the combination of metformin and gefitinib reduced proliferation in non-small-cell lung cancer 14. Guan *et al*. demonstrated that sorafenib has a significant effect on patient survival in hepatocellular carcinoma by inducing apoptosis to inhibit tumor cell proliferation 35. Wang *et al*. reported that the combination of metformin and everolimus, which is a mechanistic target of rapamycin (mTOR) inhibitor, had synergistic inhibitory effects in breast cancer 36. According to our data, although metformin and lansoprazole treatment induced AMPK phosphorylation and a decrease in cyclin D1 protein levels in the cells, the G0/G1 phase was not inhibited, and S phase expansion was observed (Figure 3). This indicates a new mechanism for cancer treatment.

The PI3K/AKT pathway is also known as the survival pathway. This pathway, when activated, promotes proliferation and survival and inhibits apoptosis 37. Romorini *et al*. demonstrated that AKT can phosphorylate GSK3β at Ser9 to inhibit GSK3β activity, thereby promoting the survival of pluripotent stem cells (PSCs) 38. The inhibition of the downstream substrates of AKT, including p21^CIP1^, p27^KIP1^, and BAD, was also indicated to increase the anti-apoptosis effect 39. Zhang *et al*. demonstrated that lansoprazole induced apoptosis in breast cancer cells in a dose-dependent manner by inhibiting intracellular proton extrusion, leading to increased intracellular ATP levels, reactive oxygen species (ROS) accumulation, and lysosomal alkalinization 23. Zhao *et al*. investigated whether lansoprazole, alone or in combination with gefitinib, induced apoptosis and G0/G1 cell cycle arrest in non-small-cell lung cancer A549 cells by inhibiting Stat3 phosphorylation, PI3K/Akt signaling, and Raf/ERK signaling 24. In the present study, we discovered that AKT phosphorylated GSK3β at Ser9 and decreased p21^CIP1^ and p27^KIP1^ protein levels; these findings imply that metformin and lansoprazole treatment tended to induce cell survival. The findings of the activation of BAD and the low expression of Bcl-2, BAX, and MCL-1 provided further evidence that the combined treatment of lansoprazole and metformin induced intrinsic apoptosis. These findings indicate the synergistic effect of lansoprazole and metformin inhibits cell growth more than it promotes cell survival.

Numerous studies have demonstrated that certain drugs interact synergistically with metformin to enhance antitumor activity in various cancer types. In these synergistic interactions, the metformin dose was between 5 mM and 30 mM 40, 41. Lansoprazole is a PPI, and a dose of approximately 0.1-1 mM was used to treat solid tumors, melanoma, osteosarcoma, glioblastoma, breast cancer, and lung cancer in in other studies 23, 24. Our study demonstrated that metformin and lansoprazole effectively inhibited the growth of gastric cancer cells at a normal serum glucose level. Furthermore, a synergistic effect was observed when 1 mM metformin and 10 μM lansoprazole were administered together. Compared with when the drugs were administered separately, the combination of the drugs decreased the drug concentration administered and reduced side effects of chemotherapeutic agents 37. Another advantage is the ability of combined anticancer agents to simultaneously target multiple molecular pathways, which reduces the possibility of tumor cells developing drug resistance. We investigated the effect of the combination of metformin and lansoprazole against gastric cancer cells. Our results demonstrated that this combination inhibits the growth of gastric cancer cells and induces apoptosis at low doses, suggesting that the combined treatment of metformin and lansoprazole can be an additional cancer treatment in clinical settings.

## Conclusion

Metformin and lansoprazole exhibit synergistic anticancer effects for AGS cells cultured in medium containing 5 mM glucose. This anticancer effect is due to the delay of proliferation and the induction of apoptosis.

## Supplementary Material

Supplementary figures, table, video legend.

Supplementary video.
