# Supplementary material for: Synergistic Effect of Metformin and Lansoprazole Against Gastric Cancer through Growth Inhibition
Source: Int J Med Sci. 2023 Apr 17;20(6):717–24. doi: 10.7150/ijms.82407 (PMC10198141; doi:10.7150/ijms.82407)
Supplement: Supplementary file 1 — Supplementary figures, table, video legend. [file ijmsv20p0717s1.pdf]

Supplementary description:

Figure S1. Viability of AGS treated with various drug concentrations. A. The AGS cell line was treated with metformin (M: 0.1–9 mM). B. The AGS cell line was treated with lansoprazole (L: 3–270 μM). C. The AGS cell line was treated with combined drugs L+M (L: lansoprazole 10 μM; M: metformin 1 mM) for 3 days and then its viability was determined using the WST-1 assay. The data was presented as the mean with SD.

Video S2. Time-lapse video. The AGS cell line was seeded in a 6-well plate (5000 cells/well). The compounds were treated for 3 days. HoloMonitor M4 captured real-time cell images, which were then converted into a video by using the HoloMonitor M4 application program.
The video file can be downloaded from the following URL. http://tdl.ibms.sinica.edu.tw/download/download.html

Figure S3. Lansoprazole and metformin exhibited a synergistic effect in AGS. The AGS cell line was treated with metformin (1 mM) and varying concentrations of lansoprazole (3, 10, 30, 90, and 270 μM), and the data was then analyzed using SynergyFinder to calculate the combination index (CI).

Figure S4. Phase-contrast photographs of the AGS cell line. The cells were treated with metformin (M; 1 mM), lansoprazole (L; 10 μM), or combined drugs L+M (L: lansoprazole 10 μM; M: metformin 1 mM) for 3 and 8 days.

Figure S5. DAPI cell cycle. The AGS cell line was treated with drugs for 3 days, stained with DAPI, and the results were analyzed using the flow cytometer and FlowJo (v7). C: control; L: lansoprazole (10 μM); M: metformin (1 mM); L+M (L: lansoprazole 10 μM; M: metformin 1 mM)

Figure S6. AGS cell line was treated with metformin (M: 1mM), lansoprazole (L: 10 μM) and L+M (L: lansoprazole 10 μM; M: metformin 1 mM) for 3 days, then using western blot to display the protein level of different drug treatment. Quantification of western-blot bands was done by ImageJ software. The data was presented as the mean with SD.

Figure S7. AGS cell line was treated with metformin (M: 1mM),

lansoprazole (L: 10 μM) and L+M (L: lansoprazole 10 μM; M: metformin 1 mM) for 3 days, then using western blot to display the protein level of different drug treatment. Quantification of western-blot bands was done by ImageJ software. The data was presented as the mean with SD.

Table S1. Antibodies used in this study

Figure S1

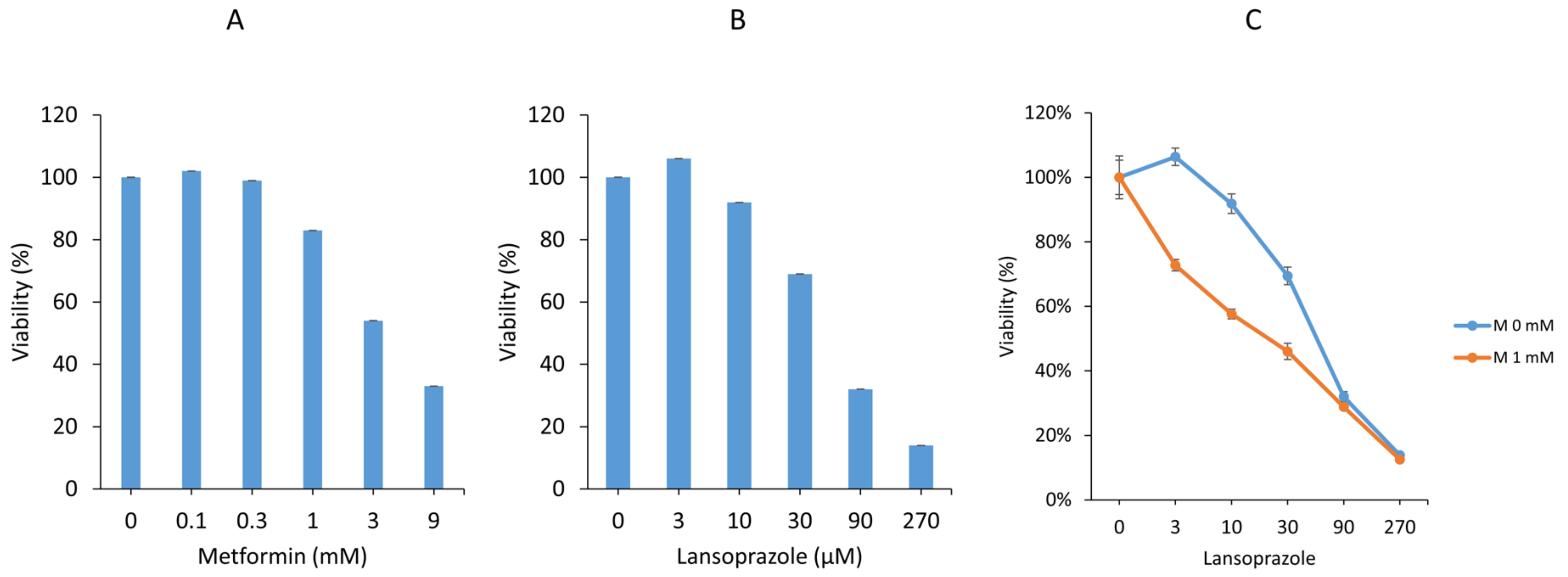
A
120
100
80
60
40
20
0
Viability (%)
0
0.1
0.3
1
3
9
Metformin (mM)
B
120
100
80
60
40
20
0
Viability (%)
0
3
10
30
90
270
Lansoprazole (µM)
C
120%
100%
80%
60%
40%
20%
0%
Viability (%)
0
3
10
30
90
270
Lansoprazole
M 0 mM
M 1 mM

Please use attached Fig2 video file

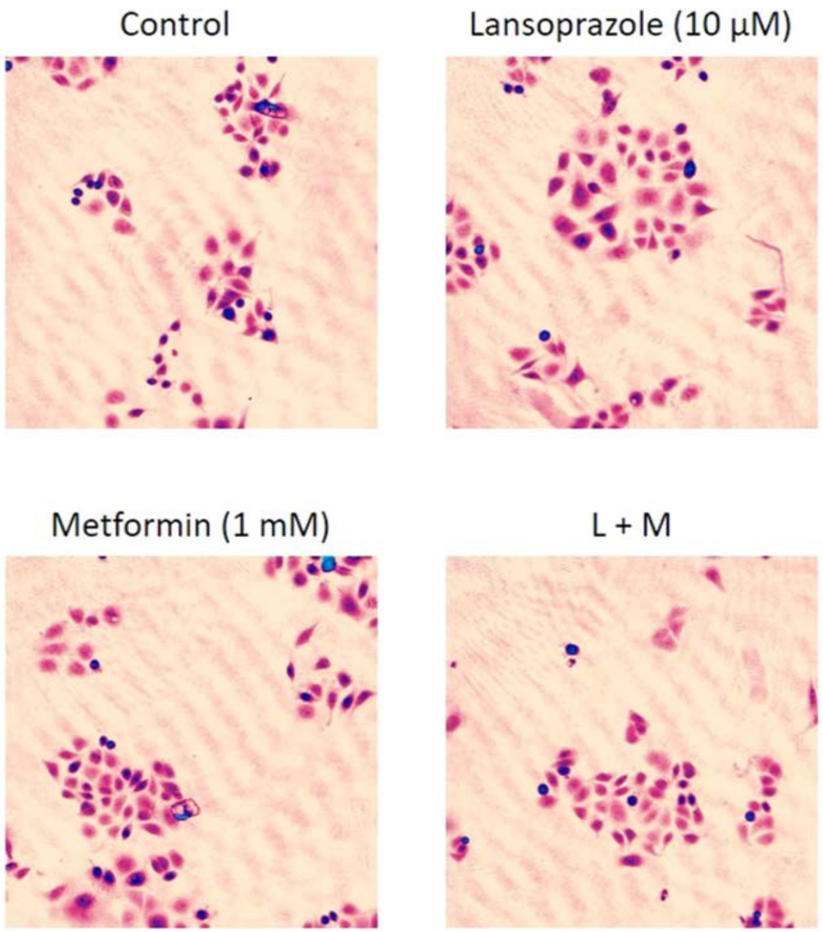

Figure S3

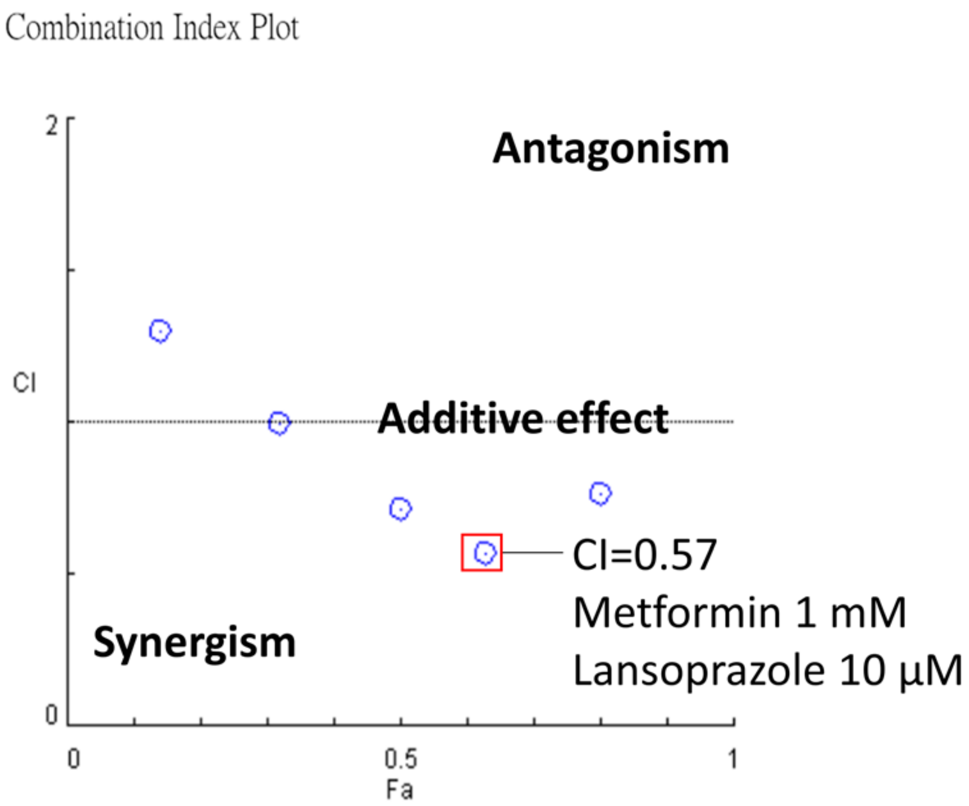
Combination Index Plot
2
CI
0
0
0.5
1
Fa
Antagonism
Additive effect
Synergism
CI=0.57
Metformin 1 mM
Lansoprazole 10 μM

Figure S4

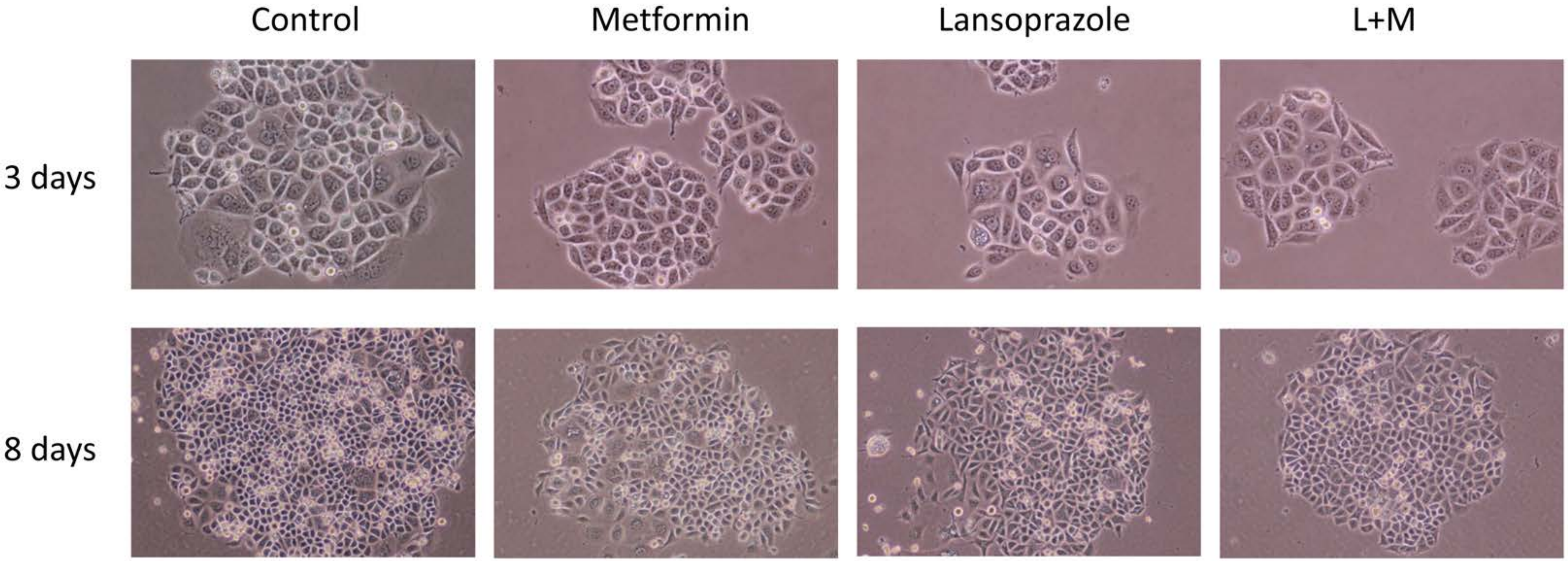

Figure S5

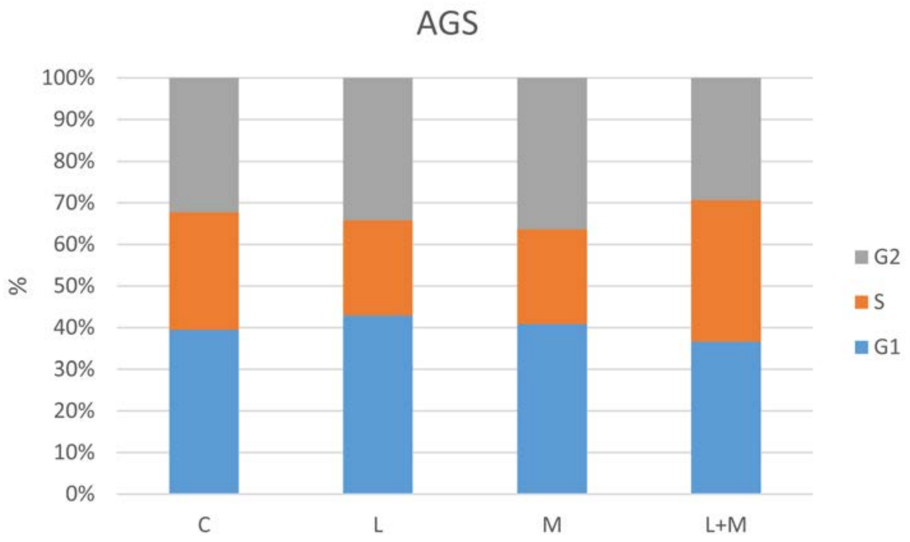

Figure S6

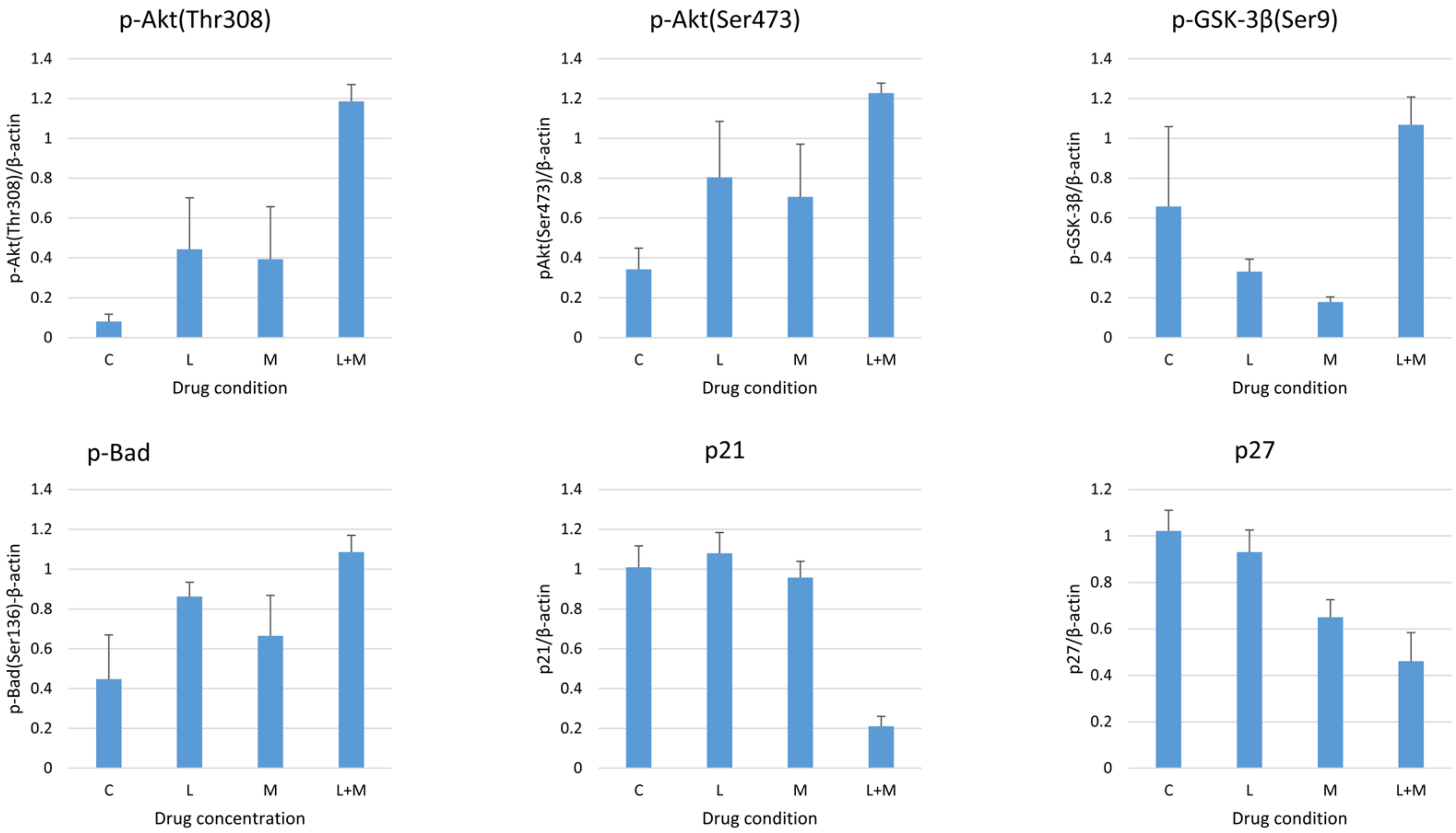
p-Akt(Thr308)
p-Akt(Thr308)/β-actin
C
L
M
L+M
Drug condition
p-Akt(Ser473)
pAkt(Ser473)/β-actin
Drug condition
p-GSK-3β(Ser9)
p-GSK-3β/β-actin
Drug condition
p-Bad
p-Bad(Ser136)-β-actin
Drug concentration
p21
p21/β-actin
Drug condition
p27
p27/β-actin
Drug condition

Figure S7

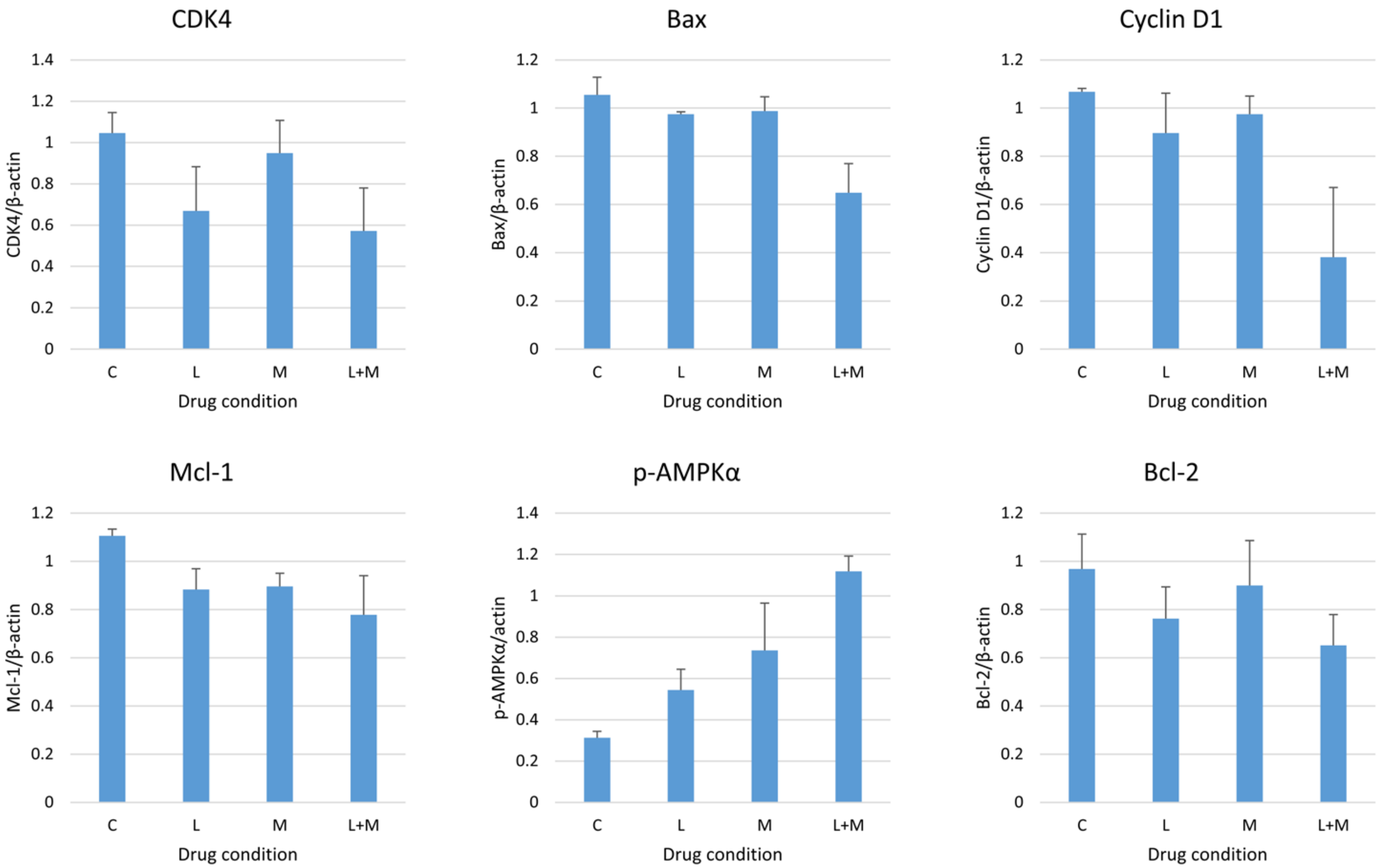

CDK4
CDK4/β-actin
1.4
1.2
1
0.8
0.6
0.4
0.2
0
C
L
M
L+M
Drug condition
Bax
Bax/β-actin
1.2
1
0.8
0.6
0.4
0.2
0
C
L
M
L+M
Drug condition
Cyclin D1
Cyclin D1/β-actin
1.2
1
0.8
0.6
0.4
0.2
0
C
L
M
L+M
Drug condition
Mcl-1
Mcl-1/β-actin
1.2
1
0.8
0.6
0.4
0.2
0
C
L
M
L+M
Drug condition
p-AMPKα
p-AMPKα/actin
1.4
1.2
1
0.8
0.6
0.4
0.2
0
C
L
M
L+M
Drug condition
Bcl-2
Bcl-2/β-actin
1.2
1
0.8
0.6
0.4
0.2
0
C
L
M
L+M
Drug condition

Table S1: Antibodies used in this study

Antibody name	1st Ab Dilution	MW (kDa)	Company	Host	2nd Ab Dilution
CDK1	1:200	34, 27	10762-1-AP, proteintech	Rabbit	1:5000
CDK4	1:1000	34	MA5-12984, ThermoFisher	Mouse	1:5000
CDK2	1:1000	34	MA5-17052, ThermoFisher	Mouse	1:5000
CyclinA2	1:500	56	18202-1-AP, proteintech	Rabbit	1:5000
CyclinB1	1:500	55-60	55004-1-AP, proteintech	Rabbit	1:5000
CyclinD1	1:200	36	MA5-16356, ThermoFisher	Rabbit	1:5000
P21	1:1000	21	#2947, Cell signaling Technology, Inc., USA	Rabbit	1:5000
P27	1:500	27	25614-1-AP, proteintech	Rabbit	1:5000
PI3K	1:1000	85	#4292, Cell signaling Technology, Inc., USA	Rabbit	1:5000
Total-AKT	1:1000	60	#4691, Cell signaling Technology, Inc., USA	Rabbit	1:5000
pAKT(Ser473)	1:1000	60	#4060, Cell signaling Technology, Inc., USA	Rabbit	1:5000
pAKT(Thr308)	1:1000	60	#4056, Cell signaling Technology, Inc., USA	Rabbit	1:5000
GSK-3β	1:1000	46	#9315, Cell signaling Technology	Rabbit	1:5000
pGSK-3β (Ser9)	1:1000	46	#9336, Cell signaling Technology	Rabbit	1:5000
Bad	1:1000	23	#9239, Cell signaling Technology, Inc., USA	Rabbit	1:5000
p-Bad (Ser136)	1:1000	23	#4366, Cell signaling Technology, Inc., USA	Rabbit	1:5000
Bcl-2	1:1000	26	#4223, Cell signaling Technology, Inc., USA	Rabbit	1:5000
Bax	1:1000	20	#5023, Cell signaling Technology	Rabbit	1:5000
Mcl-1	1:100	32, 40	sc-819, SANTA CRUZ BIOTECHNOLOGY, INC.	Rabbit	1:5000
β-actin (ACTB)	1:5000	43	MAB1501, Millipore	Mouse	1:5000

*MW: Molecular Weight
